# Plant essential oils as a novel therapeutic strategy for depression and anxiety: emerging evidence and mechanisms

**DOI:** 10.3389/fpsyt.2026.1920527

**Published:** 2026-10-08

**Authors:** Jiahui Hong, Liting He, Jingquan Chen, Hongxuan Cai, Weiyi Cheng, Ji-gang Piao, Jingjing Zhu

**Affiliations:** 1 Fuyang TCM Hospital of Orthopedics Affiliated to Zhejiang Chinese Medical University (Hangzhou Fuyang Hospital of Orthopedics of Traditional Chinese Medicine), Hangzhou, China; 2 College of Pharmaceutical Sciences, Zhejiang Chinese Medical University, Hangzhou, Zhejiang, China

**Keywords:** chemical constituents, depression and anxiety, neural pathway, plant essential oils, underlying mechanisms

## Abstract

Depression and anxiety are major global mental health disorders that require safer and more accessible adjunctive interventions. This review uniquely integrates current evidence on plant essential oils in aromatherapy and identifies the cAMP–PKA–CREB pathway as a key mechanistic hub linking neurotransmitter regulation, HPA axis modulation, neuroinflammation, and neuroplasticity. Relevant animal, cell, and preliminary clinical studies were reviewed to summarize therapeutic effects, active constituents, and commonly used essential oils. The evidence indicates that essential oils may alleviate depression- and anxiety-like symptoms by increasing monoamine neurotransmitters, suppressing HPA axis hyperactivity, reducing pro-inflammatory cytokines, inhibiting NF-κB signaling, and restoring BDNF/CREB-related neuroplasticity. Representative active constituents include linalool, β-caryophyllene, and limonene, while lavender, bergamot, and chamomile are the most commonly studied essential oils. Overall, aromatherapy shows promise as a relatively safe and practical non-pharmacological adjunct for depression and anxiety, but its evidence base remains largely preclinical. More rigorous clinical studies are needed to confirm efficacy, standardize interventions, and clarify mechanisms in humans.

## 1 Introduction

Depression and anxiety, as highly prevalent mental disorders worldwide, have posed a serious threat to individuals’ physical and mental health. Under the dual influence of the fast pace and high pressure of modern society, these disorders have shown a cross-age and cross-population pattern of penetration, continuously impairing patients’ quality of life, social functioning, and long-term well-being. Clinical studies have shown that depression and anxiety are not only common comorbidities among patients with cancer, but also reach a peak incidence among women during pregnancy and the perinatal period (1). The main clinical manifestations include depressed mood, loss of interest, cognitive impairment, and multisystem somatic symptoms, while severe cases may be accompanied by risks of self-harm or suicide. In view of this, the prevention and treatment of depression and anxiety have become priority research directions in the field of global public health in the 21st century (1).

At present, the main clinical treatments for depression and anxiety include pharmacotherapy, psychological intervention, and physical therapy. However, the use of antidepressant and anxiolytic medications still has many limitations, such as slow onset of action, a tendency to induce drug dependence, high cost, and numerous adverse effects (2). Aromatherapy is a therapeutic approach that uses essential oils extracted from natural plants and their aromas to regulate physical and psychological states through methods such as olfactory inhalation or topical application. This therapy has advantages including rapid metabolism, strong permeability, lack of retention in the body, and relatively few adverse reactions, and it has therefore attracted increasing attention from researchers and clinical practitioners. In recent years, as a non-pharmacological intervention, this therapy has been applied to the management of emotional problems such as depression and anxiety and has demonstrated positive effects (3).

## 2 Literature search strategy and article selection criteria

To ensure the comprehensiveness and reliability of the reviewed literature, a systematic literature search was conducted using electronic databases, including PubMed and Web of Science. The search strategy was developed based on a combination of keywords related to essential oils, depression, anxiety, and underlying mechanisms. The main search terms included “essential oils”, “aromatherapy”, “depression”, “anxiety”, and “mechanism”.

The literature search was limited to articles published within the past 5–10 years to capture the most recent advances in the field. Articles were screened based on titles, abstracts, and full texts according to predefined inclusion criteria. Studies were included if they (1) investigated the effects of essential oils or aromatherapy interventions on depression- or anxiety-related symptoms; (2) explored potential molecular, neurobiological, or pharmacological mechanisms; and (3) were published in peer-reviewed journals. Articles unrelated to essential oils, lacking mechanistic information, or without sufficient scientific evidence were excluded. Through this screening process, the selected studies provided the basis for summarizing the therapeutic potential and mechanisms of essential oils in the management of depression and anxiety.

## 3 Mechanisms underlying the antidepressant and anxiolytic effects of essential oils

With the continuous advancement of research in neuromolecular biology, regulatory pathways associated with neuroplasticity have gradually attracted widespread attention. Among these, the cAMP-PKA-CREB signaling pathway plays an important role in the onset, progression, and treatment of depression, and may participate in the regulation of depression- and anxiety-like behaviors by modulating neurotransmitter systems, hypothalamic–pituitary–adrenal (HPA) axis function, and neuroinflammatory responses (4). Recent studies have suggested that certain plant-derived essential oils and their bioactive constituents may indirectly regulate the cAMP-PKA-CREB signaling pathway by modulating related physiological processes. These compounds have been reported to enhance the expression of key proteins associated with this pathway and increase the levels of brain-derived neurotrophic factor (BDNF) (5, 6). In turn, BDNF can activate CREB through multiple downstream signaling cascades, including the cAMP/PKA pathway, thereby potentially forming a regulatory feedback loop that promotes synaptic plasticity and contributes to the antidepressant effects of essential oils (7–10). The multi-target mechanisms underlying the antidepressant and anxiolytic effects of essential oils are illustrated in **Figure 1**.

**Figure 1:**
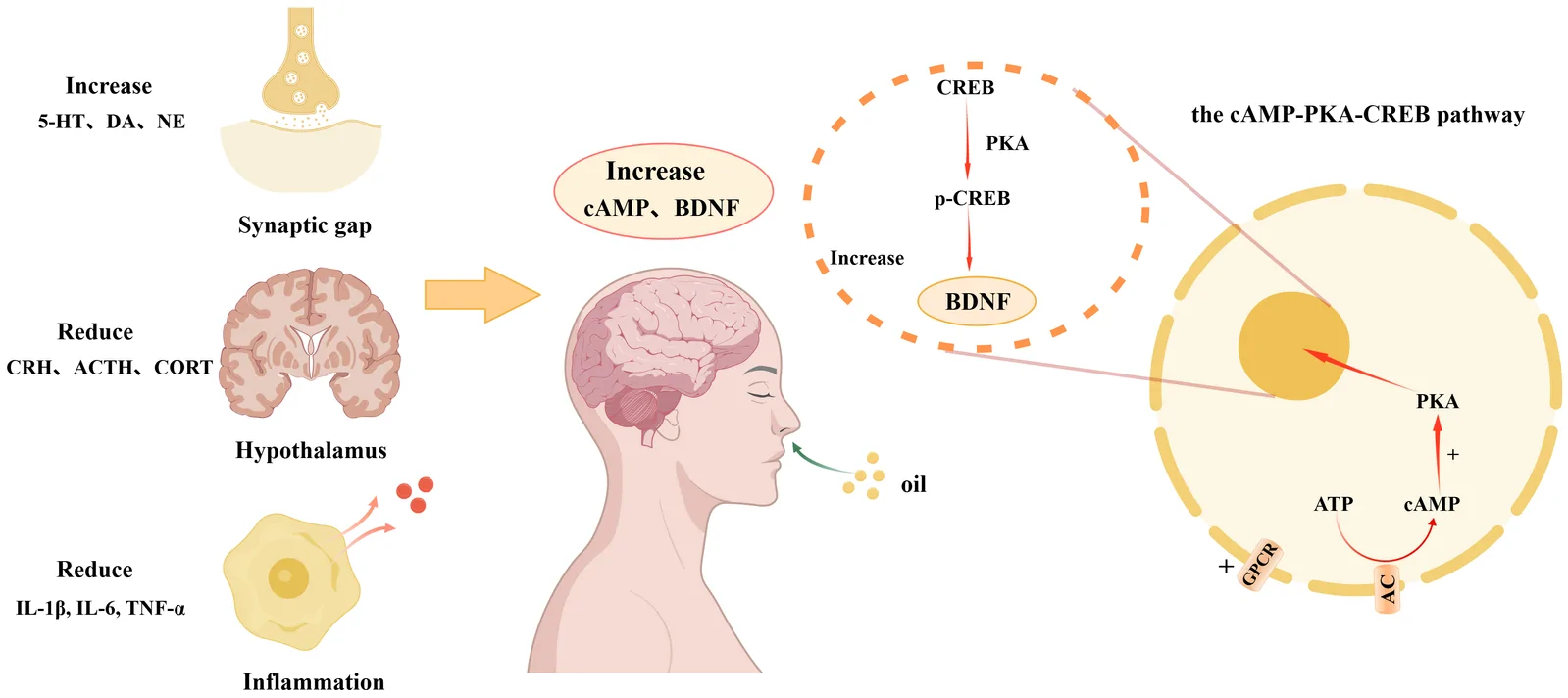
Schematic diagram illustrating molecular mechanisms of inhaled essential oils against depression and anxiety, created with BioGDP.com (20).

### 3.1 Regulatory effects on monoamine neurotransmitters (5-hydroxytryptamine, norepinephrine, and dopamine)

Monoamine neurotransmitters play an important role in the onset, progression, and treatment of depression and anxiety disorders. Previous studies have shown that patients with depression often exhibit dysfunction or abnormal levels of the above monoaminergic neurotransmitter systems (11). Therefore, most first-line antidepressants currently exert their effects primarily by regulating neurotransmitters such as 5-hydroxytryptamine (5-HT), norepinephrine (NE), and dopamine (DA) (12). For example, selective serotonin reuptake inhibitors (SSRIs) increase the concentration of 5-HT in the synaptic cleft by inhibiting the 5-HT transporter (SERT), suppressing 5-HT reuptake, and reducing its catabolism. Monoamine oxidase inhibitors (MAOIs) elevate synaptic monoamine levels by inhibiting the metabolic degradation of monoamine neurotransmitters. In addition, 5-HT3 receptor antagonists may indirectly participate in emotional regulation by modulating the excitability of relevant neural circuits (12–15). During long-term administration, these drugs can induce adaptive changes in 5-HT receptor subtypes and downstream signaling pathways, thereby positively activating the cAMP-PKA-CREB pathway and exerting antidepressant and anxiolytic effects (4). The mechanism by which antidepressants regulate serotonin (5-HT) signaling is illustrated in **Figure 2**.

**Figure 2:**
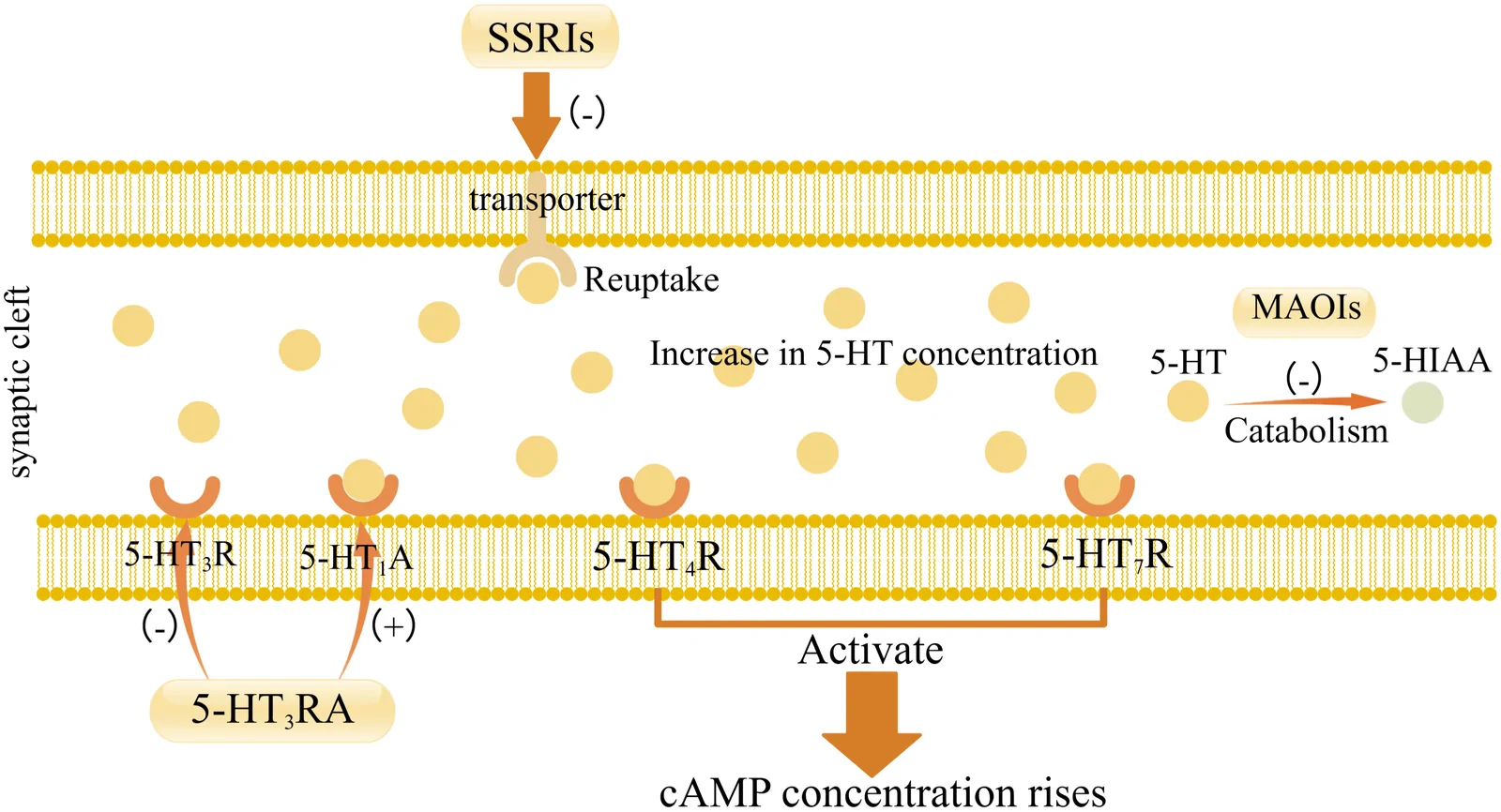
Schematic diagram of antidepressant mechanism of 5-HT-related drugs via regulating synaptic 5-HT concentration and activating downstream cAMP pathway; created with BioGDP.com (20).

Multiple animal studies have demonstrated that essential oil interventions can significantly alleviate despair-like behaviors in chronic stress-induced depression models, accompanied by increased levels of monoamine neurotransmitters, including serotonin (5-HT) and norepinephrine (NE), in the hippocampus and cortex (4, 12–15). Chen et al. (16)investigated the pathways through which saffron essential oil alleviates depression and anxiety by establishing a chronic unpredictable mild stress (CUMS) mouse model of depression. They found that saffron essential oil could exert antidepressant effects by influencing 5-HT synapses, mainly through regulating the activity of 5-HT receptors, 5-HT transporters, and monoamine oxidase inhibitors. Yu et al. (17)found, in their investigation of the therapeutic effects of Perilla frutescens essential oil (PFEO) on depression and anxiety, that inhalational administration of PFEO significantly increased the levels of 5-HT, DA, and NE in the hippocampus and cerebral cortex of CUMS rats. At a high dose, the levels of these three neurotransmitters in the cerebral cortex increased by approximately 26%, 41%, and 34%, respectively. Meanwhile, PFEO also upregulated the protein expression levels of brain-derived neurotrophic factor (BDNF) and tropomyosin receptor kinase B (TrkB), with increases of approximately 18% and 52%, respectively, in the hippocampus at the high dose. Previous studies have reported that upregulation of BDNF and TrkB expression can activate the cAMP-PKA-CREB signaling pathway, thereby contributing to antidepressant and anxiolytic effects (18). These findings suggest that the emotional regulatory effects of PFEO may be potentially associated with the modulation of the BDNF-TrkB-mediated cAMP-PKA-CREB signaling pathway (19). The study found that inhalation of CMVO significantly improved depression-like behaviors. Mechanistic analysis indicated that CMVO exerted its effects by restoring the levels of neurotransmitters such as 5-HT, DA, NE, and acetylcholine in the hippocampus, which increased by approximately 50%, 89%, 83%, and 64%, respectively, under high-dose treatment. The above studies provide preliminary and multi-source observational evidence supporting the involvement of monoaminergic systems in the antidepressant effects of essential oils. However, these findings mainly demonstrate that essential oil interventions are accompanied by alterations in monoamine neurotransmitter levels. Due to the lack of causal validation experiments, such as monoamine depletion or receptor antagonist studies, it remains unclear whether these neurotransmitter changes represent direct molecular targets of essential oils or secondary consequences resulting from stress alleviation.

### 3.2 Regulation of the hypothalamic–pituitary–adrenal axis

Hyperactivation of the HPA axis is considered a central hub in the transition from chronic stress to depression and anxiety disorders (21). Prolonged stress stimulation can lead to dysfunction of the HPA axis, which is specifically manifested as follows: chronic stress induces excessive secretion of corticotropin-releasing hormone (CRH) from the hypothalamus, which in turn drives the pituitary gland to release adrenocorticotropic hormone (ACTH), ultimately activating the adrenal cortex to secrete large amounts of corticosterone (CORT), thereby inducing depression- and anxiety-like behaviors (22). Persistent abnormal activation of the HPA axis can affect multiple intracellular neuronal signaling processes through excessive glucocorticoid release, including neuroplasticity-related pathways such as cAMP-PKA-CREB, thereby leading to reduced BDNF expression and impaired synaptic function, which further aggravate the pathological progression of depression and anxiety (23). The pathological process of HPA axis hyperactivation mediated by chronic stress and the regulatory effects of plant essential oils on this pathway are illustrated in **Figure 3**.

**Figure 3:**
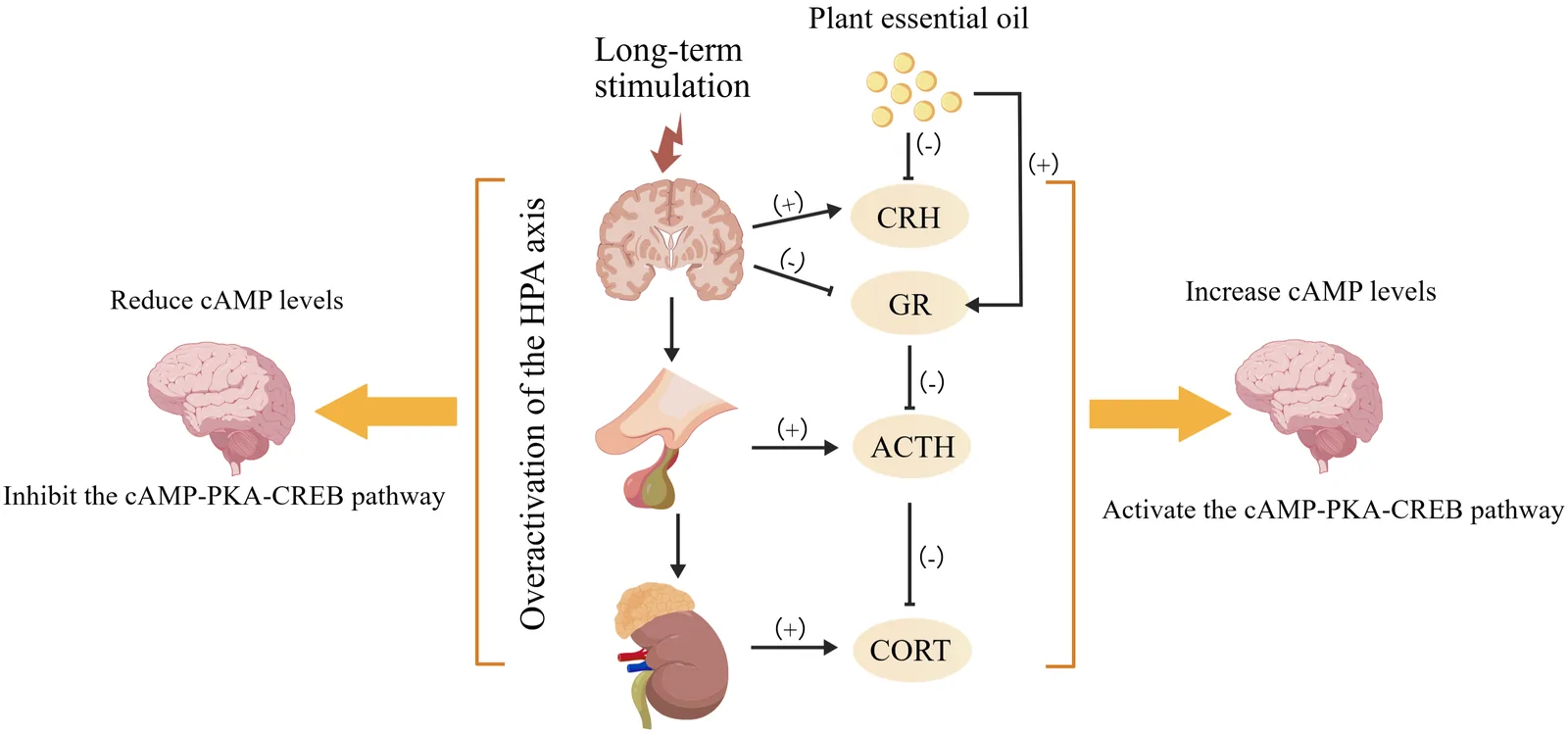
Schematic diagram of plant essential oil alleviating depression-like behavior by regulating HPA axis function and activating the cAMP-PKA-CREB pathway; created with BioGDP.com (20).

Accumulating evidence from animal studies indicates that treatment with various essential oils effectively alleviates depression-like behaviors in chronic unpredictable mild stress (CUMS) models. These behavioral improvements are associated with the normalization of HPA axis dysregulation, which may contribute to the antidepressant, anxiolytic, and neuroprotective properties of essential oils (8, 24–27). Zhang et al. (27),in their study of the therapeutic effects of Citrus aurantium essential oil (CEO) on CUMS mice, found that ELISA results showed CEO significantly reduced serum ACTH and CORT levels in CUMS mice, with decreases of approximately 24% and 18%, respectively, at the high dose. In addition, CEO increased GR expression by approximately 57% at the high dose, restored the negative feedback regulation of the HPA axis, reduced its hyperactivity, and improved depression-like behaviors in CUMS mice. The study further demonstrated that CEO could alleviate glutamate excitotoxicity and protect hypothalamic synaptic integrity by inhibiting the cAMP/PKA/Grin2b signaling pathway, thereby contributing to the restoration of HPA axis function. Tang et al. (28)treated mice with depression-like behaviors induced by chronic unpredictable mild stress (CUMS) using Zanthoxylum bungeanum fruit essential oil (HEO), and found that medium and high doses of HEO effectively ameliorated CUMS-induced depressive behaviors, with antidepressant effects comparable to those of the positive control drug sertraline hydrochloride. Further analysis showed that HEO reduced the levels of corticotropin-releasing factor (CRF) and CORT in the HPA axis, with reductions of approximately 15% and 20%, respectively, at the high dose, thereby reversing the hyperactivated state of the HPA axis and restoring its negative feedback regulation. Although the above two studies did not directly elucidate the molecular association between HPA axis restoration and activation of the cAMP-PKA-CREB pathway, their independent findings provide important clues supporting this potential link. Whether normalization of HPA axis activity creates a favorable condition for downstream cAMP-PKA-CREB pathway activation remains an intriguing scientific question that warrants further investigation. In this context, the study by Zhang et al. (8) provided more direct functional evidence. They demonstrated that CSHVO exerted neuroprotective effects by increasing cyclic adenosine monophosphate (cAMP) levels, activating protein kinase A (PKA), and promoting CREB phosphorylation. Moreover, the PKA-specific inhibitor H89 completely abolished these protective effects. These findings indicate that activation of the cerebral cAMP-PKA-CREB signaling pathway represents a key molecular mechanism underlying the ability of CSHVO to suppress neuroinflammation, protect neuronal integrity, and ameliorate CUMS-induced depressive-like behaviors in rats, providing experimental support for its potential antidepressant application in aromatherapy.

Importantly, the above studies suggest that the regulatory effects of the upstream cAMP-PKA signaling pathway within the cAMP-PKA-CREB cascade may exhibit brain region-specific characteristics in depression. In the hippocampus, PKA-mediated CREB phosphorylation and subsequent BDNF upregulation contribute to neuroprotective effects and synaptic plasticity (8). However, in the hypothalamus, excessive PKA activation may promote Grin2b phosphorylation, exacerbate glutamate excitotoxicity, and contribute to HPA axis hyperactivation (27). These findings are not contradictory; rather, they highlight that the cAMP-PKA signaling pathway may function as a context-dependent regulatory hub, exerting distinct biological effects depending on the local brain microenvironment and disease progression. This complexity should be considered when targeting the cAMP-PKA pathway for the development of antidepressant therapies.

### 3.3 Anti-inflammatory effects

Neuroinflammation is one of the important mechanisms underlying the onset and progression of depression. Under inflammatory conditions, microglia become excessively activated and release pro-inflammatory factors such as interleukin-1β (IL-1β), IL-6, and tumor necrosis factor-α (TNF-α). These inflammatory mediators can not only directly impair neuronal function, but also reduce brain cAMP levels through activation of phosphodiesterase 4 (PDE4), thereby inhibiting PKA activity and CREB phosphorylation, leading to disruption of cAMP/PKA/CREB signaling, further downregulating brain-derived neurotrophic factor (BDNF) expression, and ultimately affecting neuroplasticity and promoting the development of depression-like behaviors (29). In addition, neuroinflammation can activate NF-κB, which further suppresses CREB-related transcriptional activity and thereby aggravates neuroplasticity damage (4, 30).

Studies have shown that certain plant essential oils and their active constituents may exert antidepressant and anxiolytic effects by inhibiting central inflammatory responses, regulating NF-κB and its related downstream signals, and alleviating inflammation-mediated suppression of the BDNF/CREB pathway (8, 31, 32). Zhang et al. (32)found that Kalanchoe pinnata essential oil significantly improved depression-like behaviors in rats, accompanied by an increase in the phosphorylation level of cAMP response element-binding protein (CREB) in brain tissue by approximately 87% at the high dose, while also promoting downstream BDNF expression by about 55% at the high dose. At the same time, the study also observed reductions in serum levels of pro-inflammatory factors such as IL-1β, IL-6, and TNF-α, which decreased by approximately 46%, 30%, and 36%, respectively, under high-dose treatment. These findings suggest that Kalanchoe pinnata essential oil may improve depression-like phenotypes by simultaneously suppressing neuroinflammation and enhancing cAMP/PKA/CREB signaling. Zhao et al. (31)using a lipopolysaccharide (LPS)-induced neuroinflammatory depression mouse model and BV2 microglial cells, confirmed that Artemisia argyi–Acorus tatarinowii essential oil (CAVO) also exhibited marked anti-inflammatory and antidepressant-like effects. The results showed that CAVO reduced pro-inflammatory factors such as IL-1β, IL-6, and TNF-α in depressive mice, with reductions of approximately 24%, 12%, and 34%, respectively, at the high dose, while increasing the anti-inflammatory factor IL-10 by about 10% at the high dose. Meanwhile, cellular experiments showed that CAVO inhibited NF-κB activation by approximately 50% at the high dose and upregulated AKT-mediated CREB phosphorylation, with CREB expression increased by about 54% at the high dose, thereby further enhancing BDNF expression. These findings suggest that CAVO may exert its effects by suppressing inflammatory activation of microglia and restoring CREB/BDNF-related neuroplasticity signaling. Chowdhury et al. (33)found, in their study of the antidepressant effects of Piper betle essential oil (PBEO) and its β-cyclodextrin inclusion complex (PBECD), that both PBEO and PBECD significantly improved depression-like behaviors in mice. PBEO significantly reduced serum pro-inflammatory factors such as IL-1β and TNF-α by approximately 48.3% and 41.2%, respectively, and alleviated neuroinflammation and oxidative stress, suggesting that it may indirectly relieve inflammation-induced suppression of CREB/BDNF-related signaling by inhibiting inflammatory responses and oxidative stress.

In summary, current evidence suggests that certain plant-derived essential oils may ameliorate depression-like behaviors by suppressing neuroinflammation, reducing the release of pro-inflammatory cytokines, inhibiting NF-κB activation, and partially restoring CREB/BDNF-associated neuroplasticity signaling. However, these potential associations remain largely at the correlational level. Among the reported essential oils, Changshenghua essential oil (CSHVO) has relatively stronger evidence supporting its direct regulation of the cAMP-PKA-CREB pathway, whereas the effects of Cangai essential oil (CEO) and Piper betle essential oil are primarily characterized by the coordinated modulation between anti-inflammatory signaling and neuroplasticity-related pathways, with their direct involvement in the cAMP-PKA-CREB pathway requiring further validation. Moreover, CREB phosphorylation can be mediated by multiple upstream kinases, and the contribution of alternative signaling pathways has not been fully excluded in the aforementioned studies. Therefore, current evidence only suggests that essential oils may indirectly influence CREB/BDNF signaling through the alleviation of neuroinflammation; however, this hypothesis requires further experimental investigation for confirmation.

## 4 Antidepressant and anxiolytic constituents of plant essential oils

Plant essential oils are complex natural mixtures composed of multiple volatile constituents, and their antidepressant and anxiolytic effects are usually not determined by a single compound but rather result from the synergistic regulation of multiple components acting on multiple targets and pathways. Nevertheless, recent pharmacological and mechanistic studies have shown that some representative monomeric constituents may play key roles in the mood-regulating effects of essential oils, among which linalool, caryophyllene, and limonene have been the most extensively studied. Based on the current evidence, the following section reviews the behavioral effects and possible neurobiological mechanisms of these major active constituents.

### 4.1 Linalool

Linalool is a monoterpene compound widely present in plant essential oils and is also one of the most extensively studied active constituents with antidepressant and anxiolytic properties. Existing studies have shown that linalool can improve anxiety-like and depression-like behaviors in multiple animal models, which is closely related to its bidirectional regulation of monoamine neurotransmitter systems (34). Studies have shown that it can not only activate postsynaptic 5-HT1A receptors and enhance serotonergic signaling, thereby improving mood, but also regulate norepinephrine release through actions on α2-adrenergic receptors, thereby synergistically exerting antidepressant effects (35). This makes linalool a natural neuroactive substance with a multi-target mechanism of action (35)Liu et al. (36)found that linalool, as the main active constituent of lavender essential oil, may exert anxiolytic and sleep-promoting effects by regulating neurotransmitters and neuromodulators in the central nervous system through multiple targets. Its main targets include upregulation of NE expression by approximately 60% and downregulation of CORT levels by approximately 45%. These regulatory effects together improved insomnia-related behaviors and physiological indicators induced by the combination of anxiety and caffeine. Ying et al. (37)found that linalool directly targets the glucocorticoid receptor NR3C2, stabilizes its expression, and further upregulates the transcription of the NF-κB inhibitor zeta gene (NFKBIZ), thereby effectively suppressing activation of the NF-κB signaling pathway. According to Fonseca et al. (38)linalool mainly exerts antidepressant effects through activation of the 5-HT1A receptor. At the same time, molecular docking analysis showed that linalool also has potential binding capacity with the 5-HT2A receptor and dopamine D2 receptor, suggesting that it may regulate serotonergic and dopaminergic pathways to exert mood-regulatory functions.

### 4.2 Caryophyllene

Caryophyllene is a common sesquiterpene constituent in plant essential oils, among which the mood-regulating effects of β-caryophyllene have attracted considerable attention. It is an important functional constituent of the essential oils of clove, perilla, nutmeg, and other plants. In recent years, a large body of research has shown that this natural small-molecule compound possesses diverse biological activities (34), particularly notable anti-inflammatory, analgesic, and potential anxiolytic and antidepressant properties (39). Nguyen et al. (40)found that β-caryophyllene can act as a dual agonist of the nuclear receptor transcription factors PPARα (Peroxisome Proliferator-Activated Receptor Alpha) and PPARγ (Peroxisome Proliferator-Activated Receptor Gamma), thereby exerting antidepressant effects through multiple mechanisms such as anti-inflammatory action and neuroprotection. In addition, Nguyen et al. predicted through molecular docking that caryophyllene can bind to multiple key depression-related targets, including acetylcholinesterase (AChE), monoamine oxidase B (MAOB), SERT, dopamine transporter (DAT), norepinephrine transporter (NET), as well as inflammatory factors such as IL-1β, IL-6, and TNF-α. They further verified in *in vitro* cell models that caryophyllene can effectively alleviate neurotoxicity and neuroinflammation. Bagher et al. (41)reported that β-caryophyllene, as a selective agonist of cannabinoid receptor 2 (CB2), significantly downregulated downstream inflammatory factors TNF-α, IL-1β, and IL-6 by activating this receptor, with reductions of approximately 27%, 33%, and 34%, respectively. In this way, it effectively alleviates inflammatory responses. Li et al. (42)found that β-caryophyllene can inhibit the upstream NF-κB pathway and the core nucleotide-binding oligomerization domain-like receptor protein 3 (NLRP3) inflammasome pathway, thereby reducing the production of downstream inflammatory factors.

### 4.3 Limonene

Limonene is one of the major monoterpene constituents in many plant essential oils, especially citrus essential oils (43). Modern scientific studies have shown that limonene may exhibit potential antidepressant and anxiolytic effects through multiple pathways, including regulation of neurotransmitter systems, inhibition of neuroinflammation, and amelioration of oxidative stress, thereby providing new research ideas for the development of safe and mild natural mood interventions (44). Zhang et al. (45) in their study on the improvement of depression-like behaviors in mice by limonene, found that limonene can inhibit chronic stress-induced hyperactivity of the HPA axis and improve stress-related neuroendocrine dysfunction. It can also restore the levels of key monoamine neurotransmitters in the brain, including 5-HT and DA, and reverse the reduced expression of BDNF and its receptor in the hippocampus under depressive conditions. Alkanat et al. (46)reported that limonene can significantly reduce stress-induced elevated IL-1β levels by about 30% and restore BDNF expression. Through its anti-neuroinflammatory effects, limonene alleviates chronic stress-induced learning and memory impairment and depression-like behaviors. Eddin et al. (47) proposed that limonene exerts multidimensional neuroprotective effects by inhibiting inflammatory signaling pathways (NF-κB) and glial activation markers (Iba1, GFAP), downregulating pro-inflammatory cytokines (TNF-α,IL-1β,IL-6) iNOS, and COX-2 expression, while upregulating antioxidant enzyme activities (SOD, CAT, GSH) and neurotrophic factor expression (BDNF), and modulating apoptosis-related proteins (Bcl-2/Bax, Caspase-3) and key signaling pathways (Hippo, mTOR).

## 5 Common essential oils used for depression and anxiety and research progress

A growing number of studies have shown that essential oils play an indispensable role in antidepressant and anxiolytic interventions, among which lavender, bergamot, and chamomile essential oils are the most common. The following sections describe the principal active constituents of these three essential oils involved in antidepressant and anxiolytic effects, as well as the related research findings (**Tables 1**, **2**).

**Table 1:** Mechanisms of action of related essential oils against depression and anxiety.

Mechanism	Related essential oils	Relevant pathways
Monoamine neurotransmitters	Nutmeg essential oil, saffron essential oil, ylang-ylang essential oil, frankincense essential oil	Inhibit monoamine oxidase activity (89),and increase 5-HT and DA levels
HPA axis	Rosemary essential oil, Zanthoxylum essential oil, Zanthoxylum fruit essential oil, agarwood and Aucklandia essential oils	Lower serum corticosterone levels and inhibit the expression of corticotropin-releasing hormone, thereby regulating HPA axis function (90)
Anti-inflammatory and antioxidant effects	Eucalyptus essential oil, agarwood essential oil, bergamot essential oil, linalool essential oil	Reduce malondialdehyde (MDA), a key marker of lipid peroxidation, restore antioxidant activity, and decrease ROS levels to alleviate oxidative stres (91); inhibit pro-inflammatory pathways, reduce the levels of related pro-inflammatory factors, and enhance antioxidant pathway activity

**Table 2:** Major essential oils, bioactive constituents, molecular targets, and evidence levels in the management of depression and anxiety.

Essential oils	Active constituents	Molecular targets	Evidence levels
Lavender essential oil	Linalyl acetate and linalool	Modulation of pCREB/c-Fos signaling pathways; regulation of neurotransmitter systems (GABAergic and serotonergic pathways); improvement of gut microbiota-derived metabolites and hippocampal transcriptomic profiles	Animal studies; preliminary clinical trials
Bergamot essential oil	Limonene and other monoterpenes	Regulation of the HPA axis; modulation of serotonergic, dopaminergic, and GABAergic neurotransmission; reduction of oxidative stress and neuroinflammation through antioxidant pathways	Preclinical animal studies; preliminary human studies
Chamomile essential oil	α-Pinene and other terpenoid compounds	Enhancement of mitochondrial function; upregulation of hippocampal cellular protein-related mRNA expression; modulation of GABAergic signaling and anti-inflammatory pathways	Preclinical animal studies; randomized controlled trials in humans

### 5.1 Lavender essential oil

Lavender essential oil is currently the most commonly used essential oil for depression and anxiety and has potent sedative, relaxing, and anxiolytic properties. Lavender essential oil is mainly extracted from lavender plants by distillation or other extraction methods. Gas chromatography–mass spectrometry (GC-MS) analysis has shown that the major chemical constituents of lavender essential oil are primarily alcohols and esters, among which linalyl acetate and linalool are considered important active constituents responsible for its neuropsychological effects (48). In a study evaluating the effects of lavender essential oil on rats with depression models, Zhang et al. (49) administered an aqueous solution of lavender essential oil to the aromatherapy group by nebulized inhalation, while the control group received nebulized distilled water. The results showed that, compared with the untreated depression control group, lavender essential oil intervention significantly increased food intake, spontaneous activity, and sucrose preference in rats, suggesting that it can improve core behavioral abnormalities in animal models of depression, such as anhedonia and reduced activity, thereby exhibiting certain antidepressant- and anxiolytic-like effects. Li et al. (50) in a study of depression-like behavior in alcohol-withdrawal rats, found that rats inhaling lavender essential oil showed a significant reduction in sucrose consumption, which indicated that lavender essential oil improved the pessimistic state associated with depression. At the same time, open-field test results in that study showed that the duration of immobility was significantly reduced after lavender essential oil inhalation, indicating that lavender essential oil plays an important role in alleviating anxiety. Clinical studies have also provided some support for the application value of lavender essential oil. Yin et al. (51) randomly divided 40 patients with post-stroke depression (PSD) into an experimental group receiving lavender essential oil treatment and a control group. On the basis of conventional treatment, the antidepressant effect in the experimental group was significantly better than that in the control group, and sleep quality was also significantly improved in the experimental group. Overall, existing animal experiments and preliminary clinical studies suggest that lavender essential oil has good application prospects in alleviating depression- and anxiety-related symptoms. Its effects may be associated with the synergistic regulation of the central nervous system by active constituents such as linalool and linalyl acetate. However, current studies still have limitations in sample size, route of administration, dose standardization, and mechanistic elucidation. Future research should therefore more systematically investigate mechanisms involving neurotransmitters, the HPA axis, neuroinflammation, and neuroplasticity.

### 5.2 Bergamot essential oil

Bergamot essential oil is widely used in aromatherapy related to mood disorders and is considered to have certain antidepressant and anxiolytic potential. Its main effects are manifested in improving negative emotions, relieving anxiety, and enhancing subjective pleasure. One study using fresh bergamot for extraction and analysis showed that the main active constituents of bergamot essential oil (BEO) include monoterpene compounds such as limonene (52), which may produce pleasurable and relaxing sensations while alleviating sadness and low mood. Xu et al. (53)established a rat model of postpartum depression (PPD) using hormone-simulated pregnancy withdrawal (HSP), and after performing elevated plus maze, sucrose preference, and open-field tests, found that BEO significantly increased sucrose preference, total locomotor distance, and time spent in the open arms, while reducing time spent in the closed arms in PPD rats. These findings indicate that nebulized inhalation of BEO can significantly improve depression-like behaviors in PPD model rats. Chen et al. (54)recruited 60 women from postpartum care centers in eastern Taiwan using continuous sampling and randomly assigned them to either an experimental group receiving bergamot essential oil or a control group receiving purified water. The results showed that after two weeks of intervention, bergamot essential oil significantly improved postpartum depressive mood in women, suggesting that it may be feasible as an adjunctive intervention in routine care. In addition to postpartum populations, bergamot essential oil has also shown some regulatory effects on emotional states in generally healthy individuals. Wakui et al. (55) conducted an experiment with college students, randomly dividing them into two groups, and statistical analysis based on the Depression Anxiety Stress Scales showed that bergamot essential oil effectively improved daily mood before sleep and after waking in healthy students. This effect was also observed in healthy students confirmed by annual health examinations. These findings suggest that bergamot essential oil may not only have interventional value for clinically relevant depressive mood, but may also play a role in relieving daily stress and maintaining emotional homeostasis. Overall, current animal experiments and clinical observations indicate that bergamot essential oil has certain potential in improving depression, anxiety, and stress-related emotional symptoms. Its effects may be related to the regulation of emotion-related neural circuits by active constituents such as limonene.

### 5.3 Chamomile essential oil

In the search for complementary and alternative therapies for depression, chamomile essential oil has attracted increasing attention because of its recognized sedative and mood-regulating effects. A growing body of evidence suggests that monoterpene constituents such as α-pinene contained in chamomile essential oil may be among the important material bases for its antidepressant and anxiolytic effects (56). Roman chamomile essential oil has significant antidepressant and anxiolytic effects, mainly by improving mitochondrial function and increasing the mRNA expression of cellular proteins in the hippocampus (57). From preclinical animal experiments to randomized controlled trials in humans, current evidence consistently indicates that chamomile essential oil has significant antidepressant and anxiolytic activity, and its clinical efficacy has been preliminarily validated (57, 58). In a double-blind randomized clinical trial involving 154 patients with acute coronary syndrome (ACS), Pourshaikhian et al. (59) randomly assigned participants to a chamomile essential oil intervention group or a control group. Statistical analysis showed that the placebo group that did not receive chamomile essential oil intervention had significantly higher anxiety scores, suggesting that chamomile essential oil has a certain effect in alleviating anxiety symptoms in ACS patients. This result indicates that chamomile essential oil may not only be beneficial for primary mood disorders, but may also play an adjunctive emotional regulatory role in patients with physical illnesses accompanied by anxiety. Animal model studies have likewise provided support for its anxiolytic activity. Silveira et al. (60) exposed male and female zebrafish to different concentrations of each essential oil, and various tests and statistical analysis showed that Roman chamomile essential oil exerted anxiolytic effects, as reflected by a significant increase in the time zebrafish spent in the light zone in the light–dark box test. At a concentration of 10 mg/L, Roman chamomile essential oil significantly reduced anxiety-like behavior in adult zebrafish compared with the control group. This suggests that Roman chamomile essential oil also has stable anxiolytic-like effects in non-mammalian models, further supporting the cross-model consistency of its mood-regulating actions. Overall, current studies indicate that chamomile essential oil, especially Roman chamomile essential oil, has certain potential in anti-anxiety and antidepressant applications. Its effects may be related to improvement of mitochondrial function, regulation of hippocampus-related molecular expression, and restoration of neural functional homeostasis mediated by active constituents such as α-pinene. However, direct neurobiological evidence for its antidepressant effects remains relatively limited, and clinical studies have mainly focused on the improvement of anxiety symptoms. Therefore, its key active constituents, targets of action, and clinical scope of application still need to be further clarified.

## 6 Regulatory considerations and barriers to clinical translation

Although numerous systematic reviews and meta-analysis have investigated the therapeutic effects of essential oils on depression and anxiety, the quality of available evidence remains heterogeneous, and high-level clinical evidence is still limited. Multiple animal studies have demonstrated that essential oils exert anxiolytic and antidepressant effects through the modulation of neurotransmitter systems and signaling pathways. However, whether findings from animal models can be reliably translated to human patients requires further validation through well-designed, high-quality randomized controlled trials (RCTs). At the clinical level, the evidence remains more complex. A systematic review and meta-analysis of aromatherapy with Rosa damascena involving 28 RCTs reported that this intervention significantly alleviated anxiety symptoms (SMD = −1.31; 95% CI, −1.74 to −0.88; p < 0.001) and stress symptoms (SMD = −0.76; 95% CI, −1.07 to −0.44; p < 0.001) (61). However, its effects on depressive symptoms were relatively modest and did not reach statistical significance (p > 0.1). Syarifah Noor Syakiylla Sayed Daud et al. screened 435 studies published between 2017 and 2024 and ultimately included 45 eligible studies, indicating that although aromatherapy shows potential benefits in reducing stress, depression, and anxiety, current evidence is limited by factors such as restricted participant populations and insufficient reporting accuracy (62). Although inhalation aromatherapy has shown some potential in alleviating anxiety, depression, and insomnia, the evidence on its clinical efficacy remains inconsistent, limited by significant heterogeneity across studies, insufficient standardization of experimental methods, and inadequate quality control of essential oils. Larger, rigorously designed randomized controlled trials are needed to validate its therapeutic effects (63–65).

Beyond clinical evidence limitations, the regulatory status of plant-derived essential oils remains fundamentally ambiguous within major pharmaceutical regulatory systems, representing a primary institutional barrier to clinical translation. In the United States, essential oil products are regulated by the U.S. Food and Drug Administration (FDA) and the Federal Trade Commission (FTC). However, the lack of a clear statutory definition results in substantial classification uncertainty. Essential oil products making therapeutic claims may be considered drugs; nevertheless, many products are practically marketed and regulated as cosmetics or dietary supplements, thereby avoiding the stringent pre-market requirements imposed on pharmaceutical products, including clinical efficacy evaluation and systematic adverse event monitoring. Similar challenges exist within the European regulatory framework. Although the European Medicines Agency (EMA) provides regulatory pathways for herbal medicinal products, the complex composition and multi-component, multi-target characteristics of essential oils make it difficult to satisfy conventional registration requirements based on clearly defined active ingredients or characteristic markers. To date, only a limited number of essential oil-based products, such as the orally administered lavender oil preparation Silexan, have successfully obtained medicinal approval. While this example demonstrates the feasibility of regulatory approval for essential oil-derived medicines, it also highlights the substantial regulatory barriers faced by the majority of essential oil products.

## 7 Summary and discussion

### 7.1 Summary

This article systematically reviews the therapeutic value, mechanisms of action, and commonly used essential oils in aromatherapy interventions for depression and anxiety, with the aim of providing a scientific basis and practical reference for the non-pharmacological treatment of these conditions. Based on the existing body of research, the following core conclusions can be drawn:

First, aromatherapy offers several advantages, including rapid metabolism, strong skin permeability, low tendency for accumulation in the body, relatively low cost, and mild adverse effects. To some extent, it may compensate for the limitations of conventional antidepressants, such as delayed onset of action, potential dependence, and prominent side effects. Therefore, essential oils represent a promising non-pharmacological intervention, particularly for individuals with poor drug tolerance, those requiring long-term emotional regulation, and those in special physiological conditions such as the postpartum period or with comorbid cancer.

Second, the mechanisms by which essential oils alleviate depression and anxiety have been supported by multidimensional research, among which the cAMP-PKA-CREB pathway serves as an important shared molecular target underlying their antidepressant and anxiolytic effects. Studies have shown that essential oils may increase the levels of monoamine neurotransmitters such as serotonin, norepinephrine, and dopamine; reduce the levels of corticotropin-releasing hormone, adrenocorticotropic hormone, and corticosterone caused by hyperactivation of the hypothalamic-pituitary-adrenal axis; or inhibit abnormal activation of the PDE4/NF-κB pathway induced by pro-inflammatory factors such as interleukin-1β and tumor necrosis factor-α. Through these mechanisms, essential oils can directly or indirectly restore the activity of the cAMP-PKA-CREB pathway, upregulate brain-derived neurotrophic factor expression, improve synaptic plasticity, and thereby alleviate depression-like and anxiety-like behaviors. These findings suggest that the cAMP-PKA-CREB pathway may serve as a key functional indicator for evaluating the antidepressant and anxiolytic activities of essential oils.

In addition, this article summarizes three essential oils commonly used as adjunctive interventions for depression and anxiety, and discusses their active constituents and related clinical studies. In conclusion, existing evidence indicates that aromatherapy has a certain degree of scientific support and practical feasibility in the intervention of depression and anxiety, and may provide a safer and more readily implementable option for the adjunctive treatment of neuropsychiatric disorders.

### 7.2 Discussion

The effects of essential oils on depression and anxiety are fundamentally based on the pharmacokinetic properties of their volatile constituents, which can enter the body through inhalation or transdermal absorption. The central effects of essential oils are not solely attributed to olfactory-induced psychological perception; rather, bioactive molecules may reach the central nervous system (CNS) through systemic circulation or the nose-to-brain pathway (66, 67). However, the ability of essential oil constituents to cross the blood–brain barrier (BBB) exhibits significant structural dependency. According to a quantitative structure–activity relationship (QSAR) model developed by Snezana Agatonovic-Kustrin et al., lipophilicity (log P), molecular size, and molecular shape represent the most influential parameters for predicting BBB permeability (68). Based on the model predictions, compounds with the highest estimated brain penetration capacity were predominantly small-sized and highly lipophilic hydrocarbon terpenes within the dataset. In contrast, flavonoid components of essential oils with reported antidepressant activities generally possess relatively higher molecular weights and may have limited BBB permeability (69, 70). Under these circumstances, the nose-to-brain pathway, mediated by olfactory and trigeminal nerve transport, may bypass BBB limitations, enabling rapid brain delivery of essential oil constituents, increasing drug concentrations in brain tissues, shortening onset time, and reducing peripheral exposure, thereby representing a promising strategy for central emotional regulation (57).

Nevertheless, translational research in this field continues to face substantial challenges in standardization (71). Essential oils derived from the same plant species may exhibit distinct chemotypes due to variations in geographical origin, harvesting period, and extraction procedures, resulting in considerable batch-to-batch differences in chemical composition (72–75). Current approaches for defining essential oil doses remain inconsistent, with many studies relying on approximate measurements based on volume or diffusion duration rather than biologically relevant concentrations determined by characteristic biomarkers. Furthermore, the lack of quality control approaches, such as gas chromatography-based chemical fingerprinting, contributes to formulation variability and limits the comparability among studies, thereby hindering the establishment of reliable dose–response relationships.

The absence of standardized administration protocols represents another major barrier to evidence-based development (76). Aromatherapy interventions, including inhalation and topical application, involve diverse operational procedures, while variations in dosage units and formulations further increase intervention heterogeneity (77, 78). Moreover, no consensus has been reached regarding single-session duration, treatment frequency, or total intervention period (79). These substantial differences in treatment parameters contribute to the high heterogeneity observed in meta-analysis. Although the TREATS transparency reporting checklist provides a feasible framework for improving the standardization of aromatherapy trials, further pharmacokinetic comparisons among different administration routes are required to establish unified intervention protocols (76).

Safety evaluation also warrants careful consideration, as the assumption that “natural products are inherently safe” represents a significant misconception. Dermal exposure to essential oils may cause irritation, allergic contact dermatitis, or phototoxic reactions, whereas inhalation may exacerbate asthma or, in rare cases, induce acute eosinophilic pneumonia (80, 81). Oral administration carries potential risks of central nervous system depression and hepatic or renal injury (82). In addition, certain essential oils, including lavender and tea tree oils, have been suggested to possess potential endocrine-disrupting activities. Among different administration routes, oral use appears to be associated with a higher incidence of adverse effects.

Both inhalation and transdermal absorption can facilitate the entry of terpene compounds into systemic circulation, where they may interact with cytochrome P450 (CYP450) enzymes and alter drug metabolism (83, 84). Lavender-derived products may influence the metabolic rate of selective serotonin reuptake inhibitors (SSRIs), potentially increasing the risk of serotonin syndrome (85). Moreover, constituents such as α-pinene may share pharmacological targets with benzodiazepines, and their combined use may result in additive sedative and hypnotic effects (86–88). Therefore, interactions between essential oil constituents and psychotropic medications may present additional risks for individuals receiving psychiatric treatments.

Most studies involving non-approved essential oil interventions are characterized by small sample sizes and open-label or single-blind designs, resulting in relatively low levels of evidence and insufficient support for their therapeutic efficacy in diagnosed depressive or anxiety disorders. In contrast, approved antidepressant medications, including escitalopram, sertraline, and venlafaxine, have been rigorously evaluated through large-scale phase III randomized controlled trials and meta-analysis. These medications exert stable antidepressant and anxiolytic effects primarily through modulation of monoaminergic systems, such as serotonin (5-HT) and norepinephrine (NE), and are consistently recommended as first-line treatments for moderate-to-severe depression and various anxiety disorders in clinical guidelines worldwide.

Regarding safety, essential oils generally exhibit fewer severe adverse effects and lower risks of dependence or withdrawal compared with conventional antidepressants; however, their long-term systemic effects and potential drug interactions remain insufficiently investigated. Conversely, although antidepressants are associated with well-established adverse effects, including nausea, sexual dysfunction, weight changes, and discontinuation syndromes, their pharmacological mechanisms, metabolic pathways, and drug interactions have been extensively characterized, allowing effective risk management under clinical supervision. Therefore, non-approved essential oil products should currently be considered complementary approaches for daily emotional regulation rather than substitutes for evidence-based antidepressant medications in patients with diagnosed depressive or anxiety disorders. In particular, individuals experiencing moderate-to-severe psychological distress or suicidal risk require appropriate clinical assessment and standardized pharmacological treatment.

## References

[1] Zhang ZY Zhu YF Wang XL Chen HY . Research progress on sweet orange essential oil aromatherapy for the intervention of depression and anxiety emotions. Flavour Fragrance Cosmetics. (2024) 2:21–6. doi: 10.20099/j.issn.1000-4475.2023.0277

[2] Hu J Wang W Deng WJ . Aromatherapy with anti-depression. J Appl Technol. (2022) 22:215–24. doi: 10.3969/j.issn.2096-3424.2022.03.005

[3] Wang PY Lu WH . Aromatic substances in the treatment of depression. Chem Life. (2024) 44:581–8. doi: 10.13488/j.smhx.20230788

[4] Zixuan L Wenling P Xueling D Qing H Zeping Z Yaxuan S . Role of the Camp-Pka-Creb pathway in depression: Mechanisms and therapeutic implications. Brain Res. (2025) 1865:149895. doi: 10.1016/j.brainres.2025.149895 40840858

[5] Sánchez-Muñoz I Sánchez-Franco F Vallejo M Fernández A Palacios N Fernández M . Regulation of somatostatin gene expression by brain derived neurotrophic factor in fetal rat cerebrocortical cells. Brain Res. (2011) 1375:28–40. doi: 10.1016/j.brainres.2010.12.031 21184749

[6] Bai L Zhang S Zhou X Li Y Bai J . Brain-derived neurotrophic factor induces thioredoxin-1 expression through Trkb/Akt/Creb pathway in Sh-Sy5y cells. Biochimie. (2019) 160:55–60. doi: 10.1016/j.biochi.2019.02.011 30796965

[7] Sun K Wei X Feng P Cui M Huang S Jiang B . Neuroinflammatory mechanism of volatile oil from Acori graminei rhizoma in Tourette syndrome: Gc-Ms, network pharmacology and experimental validation. J Pharm BioMed Anal. (2025) 265:117057. doi: 10.1016/j.jpba.2025.117057 40712238

[8] Zhang S Hu Y Zhao Y Feng Y Wang X Miao M . Molecular mechanism of Chang Shen Hua volatile oil modulating brain Camp-Pka-Creb pathway to improve depression-like behavior in rats. Phytomedicine. (2024) 130:155729. doi: 10.1016/j.phymed.2024.155729 38772184

[9] Huang YJ Lu KH Lin YE Panyod S Wu HY Chang WT . Garlic essential oil mediates acute and chronic mild stress-induced depression in rats via modulation of monoaminergic neurotransmission and brain-derived neurotrophic factor levels. Food Funct. (2019) 10:8094–105. doi: 10.1039/c9fo00601j 31735946

[10] Pardossi S Fagiolini A Cuomo A . Variations in Bdnf and their role in the neurotrophic antidepressant mechanisms of ketamine and esketamine: A review. Int J Mol Sci. (2024) 25:13098. doi: 10.3390/ijms252313098 39684808 PMC11642200

[11] K SVA Verma AK A SS Sinha SS Boyina R Kosanam S . Neurotransmitter dysregulation in depression, anxiety, and suicidality: From synaptic dysfunction to cellular pathogenesis. Psychiatry Res. (2026) 361:117120. doi: 10.1016/j.psychres.2026.117120 41924767

[12] Bhatt S Devadoss T Manjula SN Rajangam J . 5-Ht(3) receptor antagonism a potential therapeutic approach for the treatment of depression and other disorders. Curr Neuropharmacol. (2021) 19:1545–59. doi: 10.2174/1570159x18666201015155816 33059577 PMC8762176

[13] Walter HJ Bukstein OG Abright AR Keable H Ramtekkar U Ripperger-Suhler J . Clinical practice guideline for the assessment and treatment of children and adolescents with anxiety disorders. J Am Acad Child Adolesc Psychiatry. (2020) 59:1107–24. doi: 10.1016/j.jaac.2020.05.005 32439401

[14] Walker FR . A critical review of the mechanism of action for the selective serotonin reuptake inhibitors: Do these drugs possess anti-inflammatory properties and how relevant is this in the treatment of depression? Neuropharmacology. (2013) 67:304–17. doi: 10.1016/j.neuropharm.2012.10.002 23085335

[15] Sun X Li N Zhong P Chen L Sun J . Development of Mao-a and 5-Ht(2a)R dual inhibitors with improved antidepressant activity. J Med Chem. (2022) 65:13385–400. doi: 10.1021/acs.jmedchem.2c01271 36173886

[16] Chen Z Gu J Lin S Xu Z Xu H Zhao J . Saffron essential oil ameliorates Cums-induced depression-like behavior in mice via the Mapk-Creb1-Bdnf signaling pathway. J Ethnopharmacol. (2023) 300:115719. doi: 10.1016/j.jep.2022.115719 36126781

[17] Zhong Y Du Q Wang Z Zheng Q Yang M Hu P . Antidepressant effect of Perilla frutescens essential oil through monoamine neurotransmitters and Bdnf/Trkb signal pathway. J Ethnopharmacol. (2024) 318:116840. doi: 10.1016/j.jep.2023.116840 37355083

[18] Gupta AK Gupta S Mehan S Khan Z Das Gupta G Narula AS . Exploring the connection between Bdnf/Trkb and Ac/Camp/Pka/Creb signaling pathways: Potential for neuroprotection and therapeutic targets for neurological disorders. Mol Neurobiol. (2025) 62:14627–59. doi: 10.1007/s12035-025-05001-5 40342191

[19] Li H Li Y Zhang X Ren G Wang L Li J . The combination of Aquilaria sinensis (Lour.) Gilg and Aucklandia costus Falc. volatile oils exerts antidepressant effects in a Cums-induced rat model by regulating the Hpa axis and levels of neurotransmitters. Front Pharmacol. (2020) 11:614413. doi: 10.3389/fphar.2020.614413 33716727 PMC7943885

[20] Jiang S LH Zhang L Mu W Zhang Y Chen T . Generic diagramming platform (Gdp): A comprehensive database of high-quality biomedical graphics. Nucleic Acids Res. (2025) 53:D1670–6. doi: 10.1093/nar/gkae973 39470721 PMC11701665

[21] Tafet GE Nemeroff CB . Pharmacological treatment of anxiety disorders: The role of the Hpa axis. Front Psychiatry. (2020) 11:443. doi: 10.3389/fpsyt.2020.00443 32499732 PMC7243209

[22] Feng X Jia M Cai M Zhu T Hashimoto K Yang JJ . Central-peripheral neuroimmune dynamics in psychological stress and depression: Insights from current research. Mol Psychiatry. (2025) 30:4881–98. doi: 10.1038/s41380-025-03085-y 40610703 PMC12436188

[23] Yang L Zhang XY Zhang HM . Antidepressant-like effects of paroxetine via regulation of Hpa axis function and activation of the Camp/Pka/Creb/Bdnf pathway in mice subjected to Cums (chronic unpredictable mild stress). Chin J Cell Biol. (2026) 48:1090–9. doi: 10.11844/cjcb.2026.04.0015

[24] Wei D Zhao Y Zhang M Zhu L Wang L Yuan X . The volatile oil of Zanthoxylum bungeanum pericarp improved the hypothalamic-pituitary-adrenal axis and gut microbiota to attenuate chronic unpredictable stress-induced anxiety behavior in rats. Drug Des Devel Ther. (2021) 15:769–86. doi: 10.2147/dddt.S281575 33654382 PMC7910097

[25] Wang S Wang C Yu Z Wu C Peng D Liu X . Agarwood essential oil ameliorates restrain stress-induced anxiety and depression by inhibiting Hpa axis hyperactivity. Int J Mol Sci. (2018) 19:3468. doi: 10.3390/ijms19113468 30400578 PMC6274913

[26] Lee MS Park WS Kim YH Kwon SH Jang YJ Han D . Antidepressant-like effects of Cortex Mori Radicis extract via bidirectional phosphorylation of glucocorticoid receptors in the hippocampus. Behav Brain Res. (2013) 236:56–61. doi: 10.1016/j.bbr.2012.08.028 22940457

[27] Zhang ZY Liu YF Zhang SJ Zhang PP Shen XX Lan JL . Rescue of Cums-induced Hpa axis hyperfunction and hypothalamic synaptic deficits by Citrus aurantium L. cv. Daidai essential oil via the Camp/Pka/Grin2b pathway. J Ethnopharmacol. (2025) 343:119423. doi: 10.1016/j.jep.2025.119423 39894418

[28] Tang D Liang Q Zhang M Li M Zhang Q Zhang S . Anti-depression effectiveness of essential oil from the fruits of Zanthoxylum bungeanum Maxim. on chronic unpredictable mild stress-induced depression behavior in mice. Front Pharmacol. (2022) 13:999962. doi: 10.3389/fphar.2022.999962 36204228 PMC9530639

[29] Ghosh M Aguirre V Wai K Felfly H Dietrich WD Pearse DD . The interplay between cyclic Amp, Mapk, and Nf-Kb pathways in response to proinflammatory signals in microglia. BioMed Res Int. (2015) 2015:308461. doi: 10.1155/2015/308461 25722974 PMC4334621

[30] Li C Chen T Zhou H Feng Y Hoi MPM Ma D . Bhdpc is a novel neuroprotectant that provides anti-neuroinflammatory and neuroprotective effects by inactivating Nf-Kb and activating Pka/Creb. Front Pharmacol. (2018) 9:614. doi: 10.3389/fphar.2018.00614 29988625 PMC6027181

[31] Zhao L Yin R Zhang K Huang J Zheng R Zhu Y . Cang-Ai volatile oil mitigates lipopolysaccharide-induced depressive-like behaviour in mice by modulating the microglial Bdnf/Creb signaling pathway. Mol Neurobiol. (2025) 63:188. doi: 10.1007/s12035-025-05325-2 41299105 PMC12657534

[32] Zhang S Chen X Wang C Sun Y Gong B Li D . Antidepressant activity of agarwood essential oil: A mechanistic study on inflammatory and neuroprotective signaling pathways. Pharm (Basel). (2025) 18:255. doi: 10.3390/ph18020255 40006068 PMC11859912

[33] Chowdhury SR Ghosh S Basu B Sen A Digar A Halder A . Deciphering the antidepressant potential of Piper betle L. essential oil and its inclusion complex: Network pharmacology and experimental insights. Mol Neurobiol. (2025) 62:13390–412. doi: 10.1007/s12035-025-05117-8 40524107

[34] Zhang Y Long Y Yu S Li D Yang M Guan Y . Natural volatile oils derived from herbal medicines: A promising therapy way for treating depressive disorder. Pharmacol Res. (2021) 164:105376. doi: 10.1016/j.phrs.2020.105376 33316383

[35] Dos Santos ÉRQ Maia JGS Fontes-Júnior EA do Socorro Ferraz Maia C . Linalool as a therapeutic and medicinal tool in depression treatment: A review. Curr Neuropharmacol. (2022) 20:1073–92. doi: 10.2174/1570159x19666210920094504 34544345 PMC9886818

[36] Liu F Wang WC Xu Y Yao L Han TT Yang SZ . Principal component analysis and functional evaluation of two essential oils based on lavender. Tianjin Agric Sci. (2023) 29:63–70. doi: 10.3969/j.issn.1006-6500.2023.04.013

[37] Hu Y Chen S Wang Y Liu L Zhang C Chen H . Linalool targets Nr3c2 to inhibit Nf-Kb-mediated gastric cancer progression. Cell Div. (2026) 21:15. doi: 10.1186/s13008-026-00188-y 42151974 PMC13352738

[38] Fonseca ECM Ferreira LR Figueiredo PLB Maia C Setzer WN Da Silva JKR . Antidepressant effects of essential oils: A review of the past decade (2012-2022) and molecular docking study of their major chemical components. Int J Mol Sci. (2023) 24:9244. doi: 10.3390/ijms24119244 37298210 PMC10252423

[39] Alnoud M Hussain MS Rios J Franco E Mills J Nwose J . Cannabidiol and beta-caryophyllene: Chronic inflammatory pain. Pharmacol Res. (2025) 222:107987. doi: 10.1016/j.phrs.2025.107987 41120021 PMC12707322

[40] Nguyen LTH Nguyen NPK Tran KN Shin HM Yang IJ . Intranasal administration of the essential oil from Perillae folium ameliorates social defeat stress-induced behavioral impairments in mice. J Ethnopharmacol. (2024) 324:117775. doi: 10.1016/j.jep.2024.117775 38224793

[41] Bagher AM . Intraplantar β-caryophyllene alleviates pain and inflammation in Stz-induced diabetic peripheral neuropathy via Cb(2) receptor activation. Int J Mol Sci. (2025) 26:4430. doi: 10.3390/ijms26094430 40362667 PMC12072555

[42] Li WY Yang F Chen JH Ren GF . β-caryophyllene ameliorates Msu-induced gouty arthritis and inflammation through inhibiting Nlrp3 and Nf-Kb signal pathway: In silico and *in vivo*. Front Pharmacol. (2021) 12:651305. doi: 10.3389/fphar.2021.651305 33967792 PMC8103215

[43] Sun J . D-limonene: Safety and clinical applications. Altern Med Rev. (2007) 12:259–64. 18072821

[44] Liu Y Ji YH Luo KX Yan XJ Liu HN Zeng ZJ . Research status of limonene application. Pharmacol Clinics Chin Materia Med. (2021) 37:244–8. doi: 10.13412/j.cnki.zyyl.20210607.002

[45] Zhang LL Yang ZY Fan G Ren JN Yin KJ Pan SY . Antidepressant-like effect of Citrus sinensis (L.) Osbeck essential oil and its main component limonene on mice. J Agric Food Chem. (2019) 67:13817–28. doi: 10.1021/acs.jafc.9b00650 30905156

[46] Alkanat M Alkanat H . D-limonene reduces depression-like behaviour and enhances learning and memory through an anti-neuroinflammatory mechanism in male rats subjected to chronic restraint stress. Eur J Neurosci. (2024) 60:4491–502. doi: 10.1111/ejn.16455 38932560

[47] Eddin LB Jha NK Meeran MFN Kesari KK Beiram R Ojha S . Neuroprotective potential of limonene and limonene containing natural products. Molecules. (2021) 26:4535. doi: 10.3390/molecules26154535 34361686 PMC8348102

[48] Zhan YT Peng B Jiang XY Liu JW Peng XW . Analysis of the main components and efficacy of true lavender essential oil. Detergent Cosmetics Sci. (2025) 48:27–32. doi: 10.3969/j.issn.1006-7264.2025.03.006

[49] Zhang Y He F He YC Song YN . Effects of lavender essential oil on expressions of pcreb and C-Fos in hippocampus and amygdala of depression Rats. Chin Arch Traditional Chin Med. (2021) 39:194–8+285-6. doi: 10.13193/j.issn.1673-7717.2021.02.049

[50] Li X Xiao D Li C Wu T Li L Li T . Lavender essential oil alleviates depressive-like behavior in alcohol-withdrawn rats: Insights from gut metabolites and hippocampal transcriptome analysis. BioMed Pharmacother. (2024) 176:116835. doi: 10.1016/j.biopha.2024.116835 38810402

[51] Yin XJ Lin GP Wu XY Huang R Xu CJ Yao MY . Effects of lavender essential oil inhalation aromatherapy on depression and sleep quality in stroke patients: A single-blind randomized controlled trial. Complement Ther Clin Pract. (2024) 55:101828. doi: 10.1016/j.ctcp.2024.101828 38241803

[52] Chang J . The effect and mechanism of bergamot essential oil atomized inhalation on hippocampal neuroplasticity in depression model rats. Tianjin: Tianjin University of Traditional Chinese Medicine (2022).

[53] Xu H . Preliminary study on the effect and mechanism of bergamot essential oil nebulized inhalation to prevent postpartum depression. Tianjin: Tianjin University of Traditional Chinese Medicine (2023).

[54] Chen ML Chen YE Lee HF . The effect of bergamot essential oil aromatherapy on improving depressive mood and sleep quality in postpartum women: A randomized controlled trial. J Nurs Res. (2022) 30:e201. doi: 10.1097/jnr.0000000000000459 34690334

[55] Wakui N Togawa C Ichikawa K Matsuoka R Watanabe M Okami A . Relieving psychological stress and improving sleep quality by bergamot essential oil use before bedtime and upon awakening: A randomized crossover trial. Complement Ther Med. (2023) 77:102976. doi: 10.1016/j.ctim.2023.102976 37625623

[56] Zhu YX Yin SY Pi MY Gao F . Analysis of essential oil content and composition of Roman chamomile. Green Technol. (2024) 26:271–5. doi: 10.16663/j.cnki.lskj.2024.08.004

[57] Cui J Li M Wei Y Li H He X Yang Q . Inhalation aromatherapy via brain-targeted nasal delivery: Natural volatiles or essential oils on mood disorders. Front Pharmacol. (2022) 13:860043. doi: 10.3389/fphar.2022.860043 35496310 PMC9041268

[58] Ebrahimi H Mardani A Basirinezhad MH Hamidzadeh A Eskandari F . The effects of lavender and chamomile essential oil inhalation aromatherapy on depression, anxiety and stress in older community-dwelling people: A randomized controlled trial. Explore (NY). (2022) 18:272–8. doi: 10.1016/j.explore.2020.12.012 33454232

[59] Pourshaikhian M Moghadamnia MT Kazemnezhad Leyli E Shafiei Kisomi Z . Effects of aromatherapy with Matricaria chamomile essential oil on anxiety and hemodynamic indices in patients with acute coronary syndrome, 2021: A randomized controlled trial. BMC Complement Med Ther. (2024) 24:17. doi: 10.1186/s12906-023-04326-9 38172912 PMC10763144

[60] Silveira V Santos Rubio KT Poleti Martucci ME . Anxiolytic effect of Anthemis nobilis L. (Roman chamomile) and Citrus reticulata Blanco (tangerine) essential oils using the light-dark test in zebrafish (Danio rerio). J Ethnopharmacol. (2022) 298:115580. doi: 10.1016/j.jep.2022.115580 35926778

[61] Xu S Shen X Xu L Xue L Wu P Wang S . The effects of Rosa damascene aromatherapy on mood and sleep: A systematic review and meta-analysis. Front Public Health. (2025) 13:1646592. doi: 10.3389/fpubh.2025.1646592 41262694 PMC12623198

[62] Daud SNSS Sudirman R . Exploring adult mental health management through aromatherapy complementary treatment. Int J Healthcare Manage. (2024) 19:387–404. doi: 10.1080/20479700.2024.243087637339054

[63] Cao X Zhou J Liu J Chen H Zheng W . Aromatherapy in anxiety, depression, and insomnia: A bibliometric study and visualization analysis. Heliyon. (2023) 9:e18380. doi: 10.1016/j.heliyon.2023.e18380 37519641 PMC10375858

[64] Gonçalves S Marques P Matos RS . Exploring aromatherapy as a complementary approach in palliative care: A systematic review. J Palliat Med. (2024) 27:1247–66. doi: 10.1089/jpm.2024.0019 38686521

[65] Chen HB Zhang YD Qin Y Jiang HY Wang LN Gu W . Inhalation aromatherapy for the treatment of comorbid insomnia: A systematic review and meta-analysis. Front Psychiatry. (2025) 16:1485693. doi: 10.3389/fpsyt.2025.1485693 40230824 PMC11994695

[66] Perry N Perry E . Aromatherapy in the management of psychiatric disorders: Clinical and neuropharmacological perspectives. CNS Drugs. (2006) 20:257–80. doi: 10.2165/00023210-200620040-00001 16599645

[67] Fung TKH Lau BWM Ngai SPC Tsang HWH . Therapeutic effect and mechanisms of essential oils in mood disorders: Interaction between the nervous and respiratory systems. Int J Mol Sci. (2021) 22:4844. doi: 10.3390/ijms22094844 34063646 PMC8125361

[68] Agatonovic-Kustrin S Chan CKY Gegechkori V Morton DW . Models for skin and brain penetration of major components from essential oils used in aromatherapy for dementia patients. J Biomol Struct Dyn. (2020) 38:2402–11. doi: 10.1080/07391102.2019.1633408 31204906

[69] Cui Y Ursuliak Z Rupasinghe HPV . Mechanistic insights into the antidepressant potential of plant-derived flavonoids: A preclinical review. Prog Neuro-Psychopharmacol Biol Psychiatry. (2025) 143:111567. doi: 10.1016/j.pnpbp.2025.111567 41285294

[70] Mohammadnezhad P Valdés A Cokdinleyen M Mendiola JA Cifuentes A . Evaluation of the *in vitro* blood-brain barrier transport of Ferula persica L. bioactive compounds. Int J Mol Sci. (2025) 26:8017. doi: 10.3390/ijms26168017 40869338 PMC12386606

[71] Ali S Ekbbal R Salar S Yasheshwar Ali SA Jaiswal AK . Quality standards and pharmacological interventions of natural oils: Current scenario and future perspectives. ACS Omega. (2023) 8:39945–63. doi: 10.1021/acsomega.3c05241 37953833 PMC10635672

[72] Zhang T Zheng Y Fu C Yang H Liu X Qiu F . Chemical variation and environmental influence on essential oil of Cinnamomum camphora. Molecules. (2023) 28:973. doi: 10.3390/molecules28030973 36770639 PMC9919661

[73] Kim M Kim S . Climate zone of geographical origin associations with essential oil composition, yield, and chemotype distribution in Coriandrum sativum L.: A multivariate analysis of 48 global accessions. Molecules. (2026) 31:1950. doi: 10.3390/molecules31111950 42280252 PMC13257553

[74] Etri K Pluhár Z . Exploring chemical variability in the essential oils of the Thymus genus. Plants (Basel). (2024) 13:1375. doi: 10.3390/plants13101375 38794445 PMC11124942

[75] Soro LC Munier S Pelissier Y Grosmaire L Yada R Kitts D . Influence of geography, seasons and pedology on chemical composition and anti-inflammatory activities of essential oils from Lippia multiflora Mold leaves. J Ethnopharmacol. (2016) 194:587–94. doi: 10.1016/j.jep.2016.10.047 27765608

[76] Reven ME Bowles EJ Audia DD Cohen MM Joswiak DJ Kurkas Lee BA . Quality appraisal of research reporting for aromatherapy and essential oil studies in humans: Proposed checklist for "Transparent Reporting for Essential Oil and Aroma Therapeutic Studies. J Integr Complement Med. (2024) 30:469–77. doi: 10.1089/jicm.2023.0006 38010213

[77] Luan J Yang M Zhao Y Zang Y Zhang Z Chen H . Aromatherapy with inhalation effectively alleviates the test anxiety of college students: A meta-analysis. Front Psychol. (2022) 13:1042553. doi: 10.3389/fpsyg.2022.1042553 36687893 PMC9853416

[78] Yang LQ Li H . Clinical application and systematic research perspectives of aromatherapy. Flavour Fragrance Cosmetics. (2025) 05:44–49. doi: 10.20099/j.issn.1000-4475.2024.0259

[79] Her J Cho MK . Effect of aromatherapy on sleep quality of adults and elderly people: A systematic literature review and meta-analysis. Complement Ther Med. (2021) 60:102739. doi: 10.1016/j.ctim.2021.102739 34166869

[80] Vora LK Gholap AD Hatvate NT Naren P Khan S Chavda VP . Essential oils for clinical aromatherapy: A comprehensive review. J Ethnopharmacol. (2024) 330:118180. doi: 10.1016/j.jep.2024.118180 38614262

[81] Kodama T Watanabe T Mataki N Kanoh S Kichikawa Y . Acute eosinophilic pneumonia following aromatherapy with essential oil. Respir Med Case Rep. (2022) 37:101657. doi: 10.1016/j.rmcr.2022.101657 35573977 PMC9092961

[82] Batubara I Fajarwati I Sari YW Guillemin GJ Maulidya I Iryawati D . Evaluation of acute and sub-acute toxicity of Curcuma aeruginosa Roxb. essential oil in Sprague Dawley rats. Res Pharm Sci. (2025) 20:456–68. doi: 10.4103/rps.Rps_9_24 40687288 PMC12271844

[83] Mondal G Dale OR Wang YH Khan SI Khan IA Yates CR . *In vitro* metabolism and Cyp-modulating activity of lavender oil and its major constituents. Molecules. (2023) 28:755. doi: 10.3390/molecules28020755 36677813 PMC9862203

[84] Ryu JH Yu J Jeon JS Jo S Lee SM Kim H . Heterotropic activation of cytochrome P450 3a4 by perillyl alcohol. Pharmaceutics. (2024) 16:1581. doi: 10.3390/pharmaceutics16121581 39771560 PMC11676982

[85] Seet HR Rotella JA . Moderate serotonin toxicity precipitated by a possible interaction between lavender tea and sertraline. Emerg Med Australas. (2021) 33:947–8. doi: 10.1111/1742-6723.13828 34212507

[86] Yang H Woo J Pae AN Um MY Cho NC Park KD . A-Pinene, a major constituent of pine tree oils, enhances non-rapid eye movement sleep in mice through Gabaa-benzodiazepine receptors. Mol Pharmacol. (2016) 90:530–9. doi: 10.1124/mol.116.105080 27573669

[87] Diniz TC de Oliveira Júnior RG Miranda Bezerra Medeiros MA Gama ESM de Andrade Teles RB Dos Passos Menezes P . Anticonvulsant, sedative, anxiolytic and antidepressant activities of the essential oil of Annona vepretorum in mice: Involvement of gabaergic and serotonergic systems. BioMed Pharmacother. (2019) 111:1074–87. doi: 10.1016/j.biopha.2018.12.114 30841421

[88] Woo J Yang H Yoon M Gadhe CG Pae AN Cho S . 3-Carene, a phytoncide from pine tree has a sleep-enhancing effect by targeting the Gaba(a)-benzodiazepine receptors. Exp Neurobiol. (2019) 28:593–601. doi: 10.5607/en.2019.28.5.593 31698551 PMC6844839

[89] Ekeanyanwu RC Nkwocha CC Ekeanyanwu CL . Behavioural and biochemical indications of the antidepressant activities of essential oils from Monodora myristica (Gaertn) seed and Xylopia aethiopica (Dunal) fruit in rats. IBRO Neurosci Rep. (2021) 10:66–74. doi: 10.1016/j.ibneur.2021.01.001 33842912 PMC8019977

[90] Ghasemzadeh Rahbardar M Hosseinzadeh H . Therapeutic effects of rosemary (Rosmarinus officinalis L.) and its active constituents on nervous system disorders. Iran J Basic Med Sci. (2020) 23:1100–12. doi: 10.22038/ijbms.2020.45269.10541 32963731 PMC7491497

[91] Wu W Wang D Shi Y Wang Y Wu Y Wu X . 1,8-Cineole alleviates hippocampal oxidative stress in Cums mice via the Pi3k/Akt/Nrf2 pathway. Nutrients. (2025) 17:1027. doi: 10.3390/nu17061027 40290092 PMC11946047

